# Predicting Environmental Suitability for a Rare and Threatened Species (Lao Newt, *Laotriton laoensis*) Using Validated Species Distribution Models

**DOI:** 10.1371/journal.pone.0059853

**Published:** 2013-03-29

**Authors:** Amanda J. Chunco, Somphouthone Phimmachak, Niane Sivongxay, Bryan L. Stuart

**Affiliations:** 1 Department of Environmental Studies, Elon University, Elon, North Carolina, United States of America; 2 Department of Biology, National University of Laos, Vientiane, Lao PDR; 3 Department of Zoology, Kasetsart University, Bangkok, Thailand; 4 North Carolina Museum of Natural Sciences, Raleigh, North Carolina, United States of America; Smithsonian’s National Zoological Park, United States of America

## Abstract

The Lao newt (*Laotriton laoensis*) is a recently described species currently known only from northern Laos. Little is known about the species, but it is threatened as a result of overharvesting. We integrated field survey results with climate and altitude data to predict the geographic distribution of this species using the niche modeling program Maxent, and we validated these predictions by using interviews with local residents to confirm model predictions of presence and absence. The results of the validated Maxent models were then used to characterize the environmental conditions of areas predicted suitable for *L. laoensis*. Finally, we overlaid the resulting model with a map of current national protected areas in Laos to determine whether or not any land predicted to be suitable for this species is coincident with a national protected area. We found that both area under the curve (AUC) values and interview data provided strong support for the predictive power of these models, and we suggest that interview data could be used more widely in species distribution niche modeling. Our results further indicated that this species is mostly likely geographically restricted to high altitude regions (i.e., over 1,000 m elevation) in northern Laos and that only a minute fraction of suitable habitat is currently protected. This work thus emphasizes that increased protection efforts, including listing this species as endangered and the establishment of protected areas in the region predicted to be suitable for *L. laoensis*, are urgently needed.

## Introduction

One-third of the world’s amphibian species are threatened with extinction and nearly half (42%) are experiencing population declines [1]. A particular challenge in amphibian conservation is a lack of basic natural history information for many species. Nearly one-fourth of the world’s amphibian species (23%) are so poorly known [1] that they are listed as Data Deficient in the IUCN Red List of Threatened Species [2]. Data are urgently needed for these taxa to prioritize the conservation of those that are threatened.

Southeast Asia has a highly diverse but highly threatened amphibian fauna [3]. New species of amphibians continue to be rapidly discovered in Southeast Asia [4], but this fauna is threatened from a high rate of deforestation and from over-harvesting for food, medicinal purposes, and the international pet trade [3]. The most critical conservation action needed for Southeast Asian amphibians is to identify, establish, and protect areas that are important for amphibian populations to mitigate this loss of biodiversity [3].

The Lao newt (*Laotriton laoensis*) was described in 2002 from a small geographic area in northern Laos [5]. After its description, commercial collectors used the publication to find and illegally harvest the species in Laos for sale into the international pet trade, where in Japan and Europe it commanded prices equivalent to >US$200 each [6]. Since then, commercial trade networks have become established within its range in northern Laos, threatening the species with extinction [7]. The species is also threatened to a lesser degree by habitat destruction and some consumption by local residents for food and medicinal purposes [7]. Although currently assessed as Data Deficient [8], the species has been recommended for upgrading to Endangered status in the IUCN Red List of Threatened Species on the basis of its restricted range and suspected population size reduction from overharvesting [7]. The species was also recommended [7] for listing in the Convention on International Trade in Endangered Species (CITES). In 2008, the government of Laos banned commercial trade in the species, although this has been inadequately enforced [7].

A major remaining deficiency in knowledge of this species is the extent of its geographic range. *Laotriton laoensis* has been reported in the literature from only nine localities [5], [7], [9], with a verified extent of occurrence of approximately 4,800 km^2^ [7]. The species is assumed to be endemic to Laos based on its known distribution, but the eastern extent of its range is uncertain and it could potentially extend beyond the border into adjacent northern Vietnam. The species is not known to occur within any of Laos’ national protected areas [7], although several protected areas occur at the periphery of the species’ known range in northern Laos. If the species does not occur in one of these national protected areas, then a new protected area needs to be established within its range to safeguard this unique species [7]. Verification of the extent of this species’ geographic distribution is urgently needed.

Because field work is frequently resource intensive, one method for prioritizing areas for field surveys is species distribution modeling. Generally, species distribution models (SDMs) integrate observations of species occurrences with environmental variables to predict the range of the species of interest [10]. These models have been highly useful in correctly identifying suitable habitat for many species, even in understudied geographic regions [11], [12]. SDMs are increasingly important in conservation biology and have been used to discover new populations of rare and endangered species [13], prioritize areas for conservation [14], and to predict levels of gene flow between populations [15]. As the distribution of *L. laoensis* remains poorly understood, SDMs are an ideal tool for predicting locations of additional, as of yet undiscovered, populations.

In this study, we use the species distribution modeling program Maxent to predict the distribution of *L. laoensis*. We undertake this work with four goals: 1) to test the hypothesis that this species is restricted to northern Laos, 2) to determine whether or not any of the established protected areas in Laos provide suitable habitat for *L. laoensis*, 3) to characterize the environmental conditions of suitable areas to aid conservation efforts, and 4) to prioritize areas for future field surveys.

## Methods

### Ecological Niche Modeling

To identify suitable habitat for *Laotriton laoensis*, we used the ecological niche modeling program Maxent (ver. 3.3.3e, [16]). We chose Maxent over other modeling algorithms as it: 1) performs well compared to other niche modeling programs [17], 2) requires only presence data [16]), and 3) has previously been used successfully with very small sample sizes (i.e. as few as five records [12]. Maxent integrates environmental data with species locality information to give a relative measure of suitability across a study area [16]. For environmental data, we used 19 bioclimatic variables and altitude data downloaded from World Clim Global Climate Data (www.worldclim.org ver. 1.4, [18]. Thus, suitable land was identified based on climate and altitude profiles. All environmental variables were at a resolution of 1 km×1 km.

For species localities, we used nine published field localities of *L. laoensis* [5], [7], [9] (Table 1). Because the variables included in any model can affect the model outcome [19], we ran several independent models using different suites of environmental variables to determine how sensitive the model results were to the specific parameters (Table 2). We employed four strategies to select variables for each model. First, we ran a complete model using all 19 Bioclimatic variables and altitude (the Full Model). Second, we used the results of the first model to select a subset of variables that contributed at least 5% to the Full Model to include in the second model (the Reduced Model). Third, because environmental variables are often highly correlated, we selected a subset of variables that were relatively uncorrelated for the next two models. To do so, we used Pearson’s correlation coefficient (r) to measure the relative degree of correlation between each pair-wise combination of variables using environmental data extracted from 100 randomly selected points each placed within 100 km of a known *L. laoensis* locality. We selected 100 km as the threshold to ensure points were reasonably close to known localities, while still providing enough distance to encompass the range of habitats in northern Laos. We randomly selected a set of relatively uncorrelated variables to include in the model using two different correlation thresholds, r>0.75 for the ‘Moderate Correlation Model’ and r>0.5 for the ‘Low Correlation Model’. Finally, we used expert opinion; one of the authors (BLS) *a priori* selected a suite of variables likely to be most important based on his experience studying *L. laoensis* in the wild. These variables were used in the ‘Expert Opinion Model’.

**Table 1:** Localities used to model habitat suitability for *Laotriton laoensis*.

Site Use	Province	District	Stream(Houay/Nam)	Coordinates*	Elev. (m)	Source
Model construction	Vientiane	Xaysomboun	H. Pa Tin	18°52′N 103°06′E	1,160	Stuart and Papenfuss 2002; Phimmachak et al. 2012
Model construction	Vientiane	Xaysomboun	H. Sang Kat	18°52′N 103°08′E	1,400	Stuart and Papenfuss 2002; Phimmachak et al. 2012
Model construction	Vientiane	Xaysomboun	H. Sang Kat	18°52′N 103°07′E	1,240	Phimmachak et al. 2012
Model construction	Vientiane	Xaysomboun	H. Thongkham	18°45′N 103°08′E	1,350	Phimmachak et al. 2012
Additionalmodel validation	Vientiane	Xaysomboun	N. Pi	19°09′N 102°42′E	1,243	North Carolina State Museum of Natural Sciences (NCSM) 79785–86
Model construction	Xiengkhouang	Pek	H. Pieng	19°42′N 103°18′E	1,430	Phimmachak et al. 2012
Model construction	Xiengkhouang	Phoukout	H. Lueng	19°43′N 103°16′E	1,227	Stuart and Papenfuss 2002 (as “near Ban Nyot Phae”)
Model construction	Xiengkhouang	Phoukout	H. Hao, H. Pee	19°42′N 103°13′E	1,144	Goldschmidt and Koehler 2007 (as “Ban Lae Village”)
Model construction	Louangphabang	Phoukoun	H. Hin Rup	19°24′N 102°34′E	1,219	Phimmachak et al. 2012
Model construction	Louangphabang	Phoukoun	N. Madao	19°18′N 102°33′E	1,245	Phimmachak et al. 2012

* Coordinates were rounded to the nearest minute to prevent facilitating exploitation of the species, following Phimmachak et al. (2012).

**Table 2:** A description of the environmental data used in the five Maxent models.

	Full Model	Reduced Model(Regional)	Reduced Model(Local)	Moderate Correlation(r <0.75)	Low correlation(r <0.5)	Expert Opinion
**Annual Mean Temperature**	x					x
Mean Diurnal Range in Temperature	x			x		
Isothermality	x		x	x	x	
**Temperature Seasonality**	x	x	x	x		
Max Temperature of Warmest Month	x					x
Min Temperature of Coldest Month	x					x
Temperature Annual Range	x					
Mean Temperature of Wettest Quarter	x					
Mean Temperature of Driest Quarter	x					
Mean Temperature of Warmest Quarter	x	x				x
Mean Temperature of Coldest Quarter	x					x
Annual Precipitation	x	x				x
Precipitation of Wettest Month	x					
Precipitation of Driest Month	x	x				
Precipitation Seasonality	x	x		x	x	
Precipitation of Wettest Quarter	x			x		
Precipitation of Driest Quarter	x					
Precipitation of Warmest Quarter	x	x		x	x	
Precipitation of Coldest Quarter	x			x	x	
Altitude	x		x	x	x	x

The same environmental parameters were used at the Full, Moderate Correlation, Low Correlation, and Expert Opinion models at both the Regional and the Local scale. For the Reduced Model, which was based on the results of the Full Model, a different suite of parameters was used at each scale as indicated below.

In selecting the geographic extent of any Maxent model, the background points chosen can strongly affect model outcome [20]. As we were interested in both the total predicted extent of the range and identifying other areas where *L. laoensis* is most likely to be found in the vicinity of where the species is known to occur, we ran each model at two different geographic scales. At the regional scale, the study area encompassed the entire Indochinese peninsula. Predicting across this entire area allowed us to determine the likelihood that the range of *L. laoensis* extended beyond the current observed range in northern Laos. At the local scale, the study area included only northern Laos and adjacent parts of Thailand and Vietnam. By drawing background points from this much smaller points, we were able to created more refined predictions without extrapolating predictions to areas well outside the likely range of the species (e.g. [20]).

Four of the models (the Full Model, Moderate Correlation, Low Correlation, and Expert Opinion) used the same set of environmental variables at both the Regional and Local scale. In the fifth model (i.e., the Reduced Model), we used a different set of parameters at each scale (Table 2) because the parameters used in the Reduced Model were specifically chosen based on the results of the Full Model, and these results differed at each scale (Table 2).

Under the logistic output setting, Maxent returns a grid where each location in the study area is assigned a value between 0 and 1, where 0 represents a low probability of occurrence (i.e. low habitat suitability), and 1 represents a high probability of occurrence (i.e. high habitat suitability) [21]. To visualize these results, we projected the Maxent output for each model in ArcGIS 9.3 using a UTM 48 N projection. All models were run nine times using the cross validation re-sampling method. Specifically, we ran a jackknife procedure with eight of the nine localities being used to train the model, with a different locality left out in successive iterations of the model [12]. The average of these nine runs was used in all analyses.

We used the default values of MAXENT with one exception: for the Full model, we further tested two alternative regularization multipliers (0.5 and 5) in addition to the default value of 1 to determine whether adjusting the regularization parameter would improve model performance ([21]).

To compare between the different models at each spatial scale, we calculated the pairwise correlations coefficient between each pair of models. To do so, we extracted the logistic value from 5000 random points throughout the area modeled at the regional spatial scale, and 1000 random points at the local spatial scale. We then calculated the Pearson’s correlation coefficient (r) of the logistic values between each pair of models.

All necessary permits were obtained for the described field studies. The Nam Ngum 3 Power Company Limited provided permission for fieldwork. The CITES Management Authority, Department of Forestry, Ministry of Agriculture and Forestry, Vientiane, Laos, provided a specimen export permit (No. 035/12) to the North Carolina State Museum of Natural Sciences, and the U.S. Fish and Wildlife Service cleared importation into the United State (eDec 2012991011). Live vertebrates were handled in accordance with the Institutional Animal Care and Use Committee of the North Carolina State Museum of Natural Sciences Protocol No. NCSM 2011-01.

### Model Validation

To validate the models, we compared the results of the Maxent model run using only field localities to presence and absence data available from interviewing local residents. *Laotriton laoensis* is distinctive, diurnal, and frequently encountered by rural people in Laos who use the streams in which it occurs to fish and extract natural resources. Thus, the species is usually well known to people who live in the same geographic area. Local residents at each survey locality were shown life-sized color photographs exhibiting dorsal and ventral views of live *L. laoensis* and asked whether they knew this animal [7]. Those who recognized it were asked where it lives, how it moves, and to estimate its size in order to verify positive responses. At 13 sites, interviewees reported that the species was present, while at 23 sites, interviews were unfamiliar with the species [7].

To compare the model results with interview data, we converted the continuous logistic output to a binomial classification of either ‘suitable’ or ‘unsuitable’ land using a threshold based on the model results. We selected the ‘lowest presence threshold’ as the threshold for differentiating habitat types. The lowest presence threshold is the smallest logistic value associated with one of the observed species localities [12]. Any area with a logistic value above the threshold was considered suitable, while any area with a value below the threshold was considered unsuitable. This threshold (like all threshold approaches) is admittedly arbitrary. This threshold was, however, relatively conservative [12] and excluded a significant fraction of the study area while still categorizing each of the nine known localities as occurring in suitable habitat.

We then used this binomial classification to determine whether each of the 36 interview sites occurred in suitable or unsuitable areas. Each interview site thus fell into one of four categories: 1) Maxent correctly predicted the presence of *L. laoensis* (i.e., true presence), 2) Maxent correctly predicted absence of *L. laoensis* (i.e. true absence), 3) Maxent predicted presence in areas where interview data showed the species is absent (i.e., false presence) and 4) Maxent predicted absence where interview data showed presence (false absence). We used a chi-square test to determine the association between Maxent predictions and species presence or absence.

We also used the interview sites where the species was recorded as present to further validate the model by rerunning the full model at the local scale using only interview presences as locality data. In addition to the interview data, we used an additional, tenth locality record of *L. laoensis* that was discovered in Laos by SP, NS, and BLS during field surveys in May 2012 immediately following the completion of the initial Maxent modeling (Table 1). We both compared this test locality to the model predictions, and re-ran the field locality models incorporating this new site to determine whether or not it affected the model results.

### Description of Suitable Environment

A good description of suitable habitat is essential for both targeting field surveys and for conservation strategy. To broadly characterize the climatic and altitudinal conditions of areas that were identified as suitable, we used the Full Maxent model at the local scale. This model contained all the environmental data we used and was focused on the region where this species is known to occur. We evaluated the response curves generated by Maxent for each environmental variable. The response curves show the relationship between the model prediction and each variable [22].

To identify the specific individual environmental factor that was limiting, we used a new tool developed by Elith *et al.* [20]. The limiting factor map identifies the specific environmental variable that most influences that model results at any given point, providing useful data for identifying the drivers of the species distribution (see Elith et al. [20] Appendix S3 for specific information and code). We performed a limiting factor analysis on the full model results at both the regional and the local scale.

To determine whether or not any area identified as suitable overlapped with a protected area, we overlaid the binomial map of suitable and unsuitable habitat with a map of the National Protected Areas in Laos. These were obtained from Protected Planet on March 23, 2012 (www.protectedplanet.net [23]). We then used the ‘intersect’ tool from Hawth’s Tools [24] to determine whether there was any overlap between the predicted habitat and currently protected areas.

## Results

### Ecological Niche Modeling

**Figure 1:**
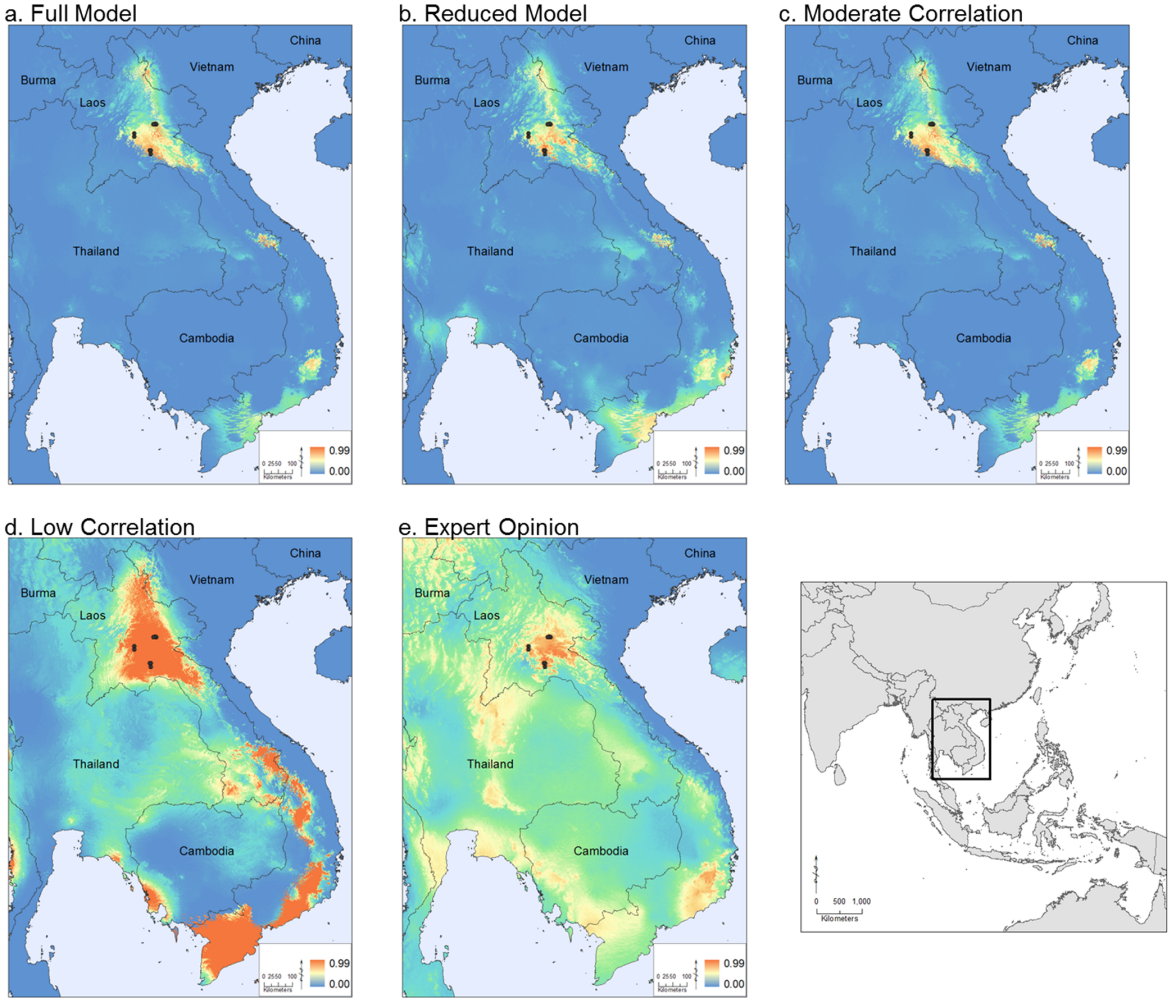
Regional Maxent model results. These results show the habitat suitability for *L. laoensis*, under five unique combinations of environmental variables: a) Full Model, b) Reduced Model, c) Moderate Correlation (r <0.75), d) Low Correlation (r <0.5), e) Expert Opinion (see Table 2). The study area is shown as the boxed area in the inset map on the lower right.

We ran several Maxent models that highlighted the climatic and altitudinal suitability for *Laotriton laoensis* using nine published field localities as species presence data. At the regional scale, all five models identified areas of high suitability in northern Laos and additional areas in southern Laos and southern Vietnam (Figure 1). The first three models (Full, Reduced, and Moderate) showed strong similarity in the areas predicted as suitable (r >0.8, Table 3). The Low Correlation and the Expert Opinion models identified a much greater extent of habitat suitability and less similarity to any of the other models (r <0.7). In particular, the Low Correlation model highlighted several areas of very high suitability. However, because this model was based on the least amount of environmental data, (i.e., only 5 of the 19 environmental layers were included because of the stringent correlation requirements), this model was the least constrained and thus it is likely that this model over-predicted suitable habitat. While the relative amount of suitable habitat differed between the models, all models showed that suitable area in northern Laos is surrounded by unsuitable habitat.

**Table 3:** The pairwise Pearson’s correlation coefficient between all pairs of models at the regional scale.

	Full Model	Reduced Model	Moderate Correlation (r<0.75)	Low Correlation(r<0.75)	Expert Opinion
Full Model		0.857	0.899	0.619	0.364
Reduced Model			0.806	0.629	0.347
Moderate Correlation (r<0.75)				.651	0.276
Low Correlation (r<0.75)					0.384
Expert Opinion					

**Figure 2:**
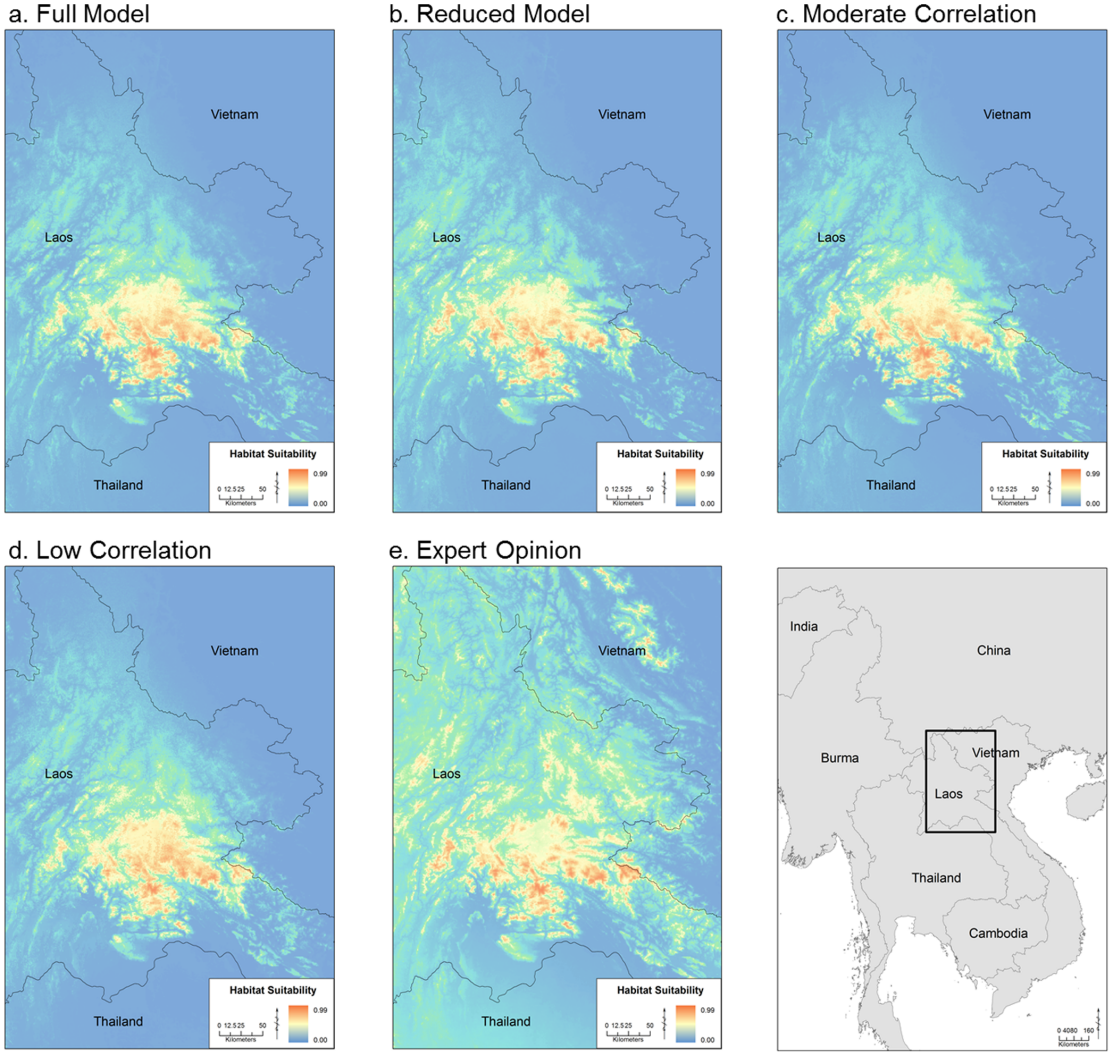
Local Maxent model results. These results show the habitat suitability for *L. laoensis*, under five unique combinations of environmental variables: a) Full Model, b) Reduced Model, c) Moderate Correlation (r <0.75), d) Low Correlation (r <0.5), e) Expert Opinion (see Table 2). The study area is shown as the boxed area in the inset map on the lower right.

For the local scale models, the areas of suitable habitat were highly similar between all five models, with all correlations above 0.945 (Figure 2, Table 4). Even the Reduced Model, with only three environmental variables, captured the same geographic extent of suitable habitat as the Full Model with 19 variables. Thus, within this more limited geographic extent, only a few key climatic variables are important in identifying suitable habitat. All models identified a limited area in northern Laos as suitable habitat (Figure 2). These models also suggested that there may be additional suitable habitat to the east of the area where this species has previously been documented.

**Table 4:** The pairwise Pearson’s correlation coefficient between all pairs of models at the local scale.

	Full Model	Reduced Model	Moderate Correlation (r<0.75)	Low Correlation(r<0.75)	Expert Opinion
Full Model		1.000	0.991	0.986	0.948
Reduced Model			0.991	0.986	0.948
Moderate Correlation (r<0.75)				0.995	0.957
Low Correlation (r<0.75)					0.962
Expert Opinion					

### Model Validation

For all models at both the regional and the local scale that were run using field locality data, the area under the curve (AUC) of the receiver operatic characteristic plot (ROC) was quite high (at least 0.969, Table 5). As AUC values above 0.75 are considered informative ([21]) our uniformly high values indicate that all models provided good discrimination between true positives and false positives [25], [26]. The default parameters performed well; adjusting the regularization parameter resulted only slight differences in AUC for the full model at the local scale (AUC of 0.970 and 0.967 for regularization parameters of 0.5 and 5 respectively) and resulted in the same order of percent contribution of variables for all variables contributing at least 5% to the model. Thus, all results presented are from models using the default parameters.

**Table 5:** The mean area under the curve (AUC) for each Maxent model at both the regional and local scale.

	Regional Models AUC	Local Models AUC
Full Model	0.997	0.969
Reduced Model	0.996	0.970
Moderate Correlation (r<0.75)	0.998	0.970
Low Correlation (r<0.75)	0.989	0.969
Expert Opinion	0.993	0.970

**Figure 3:**
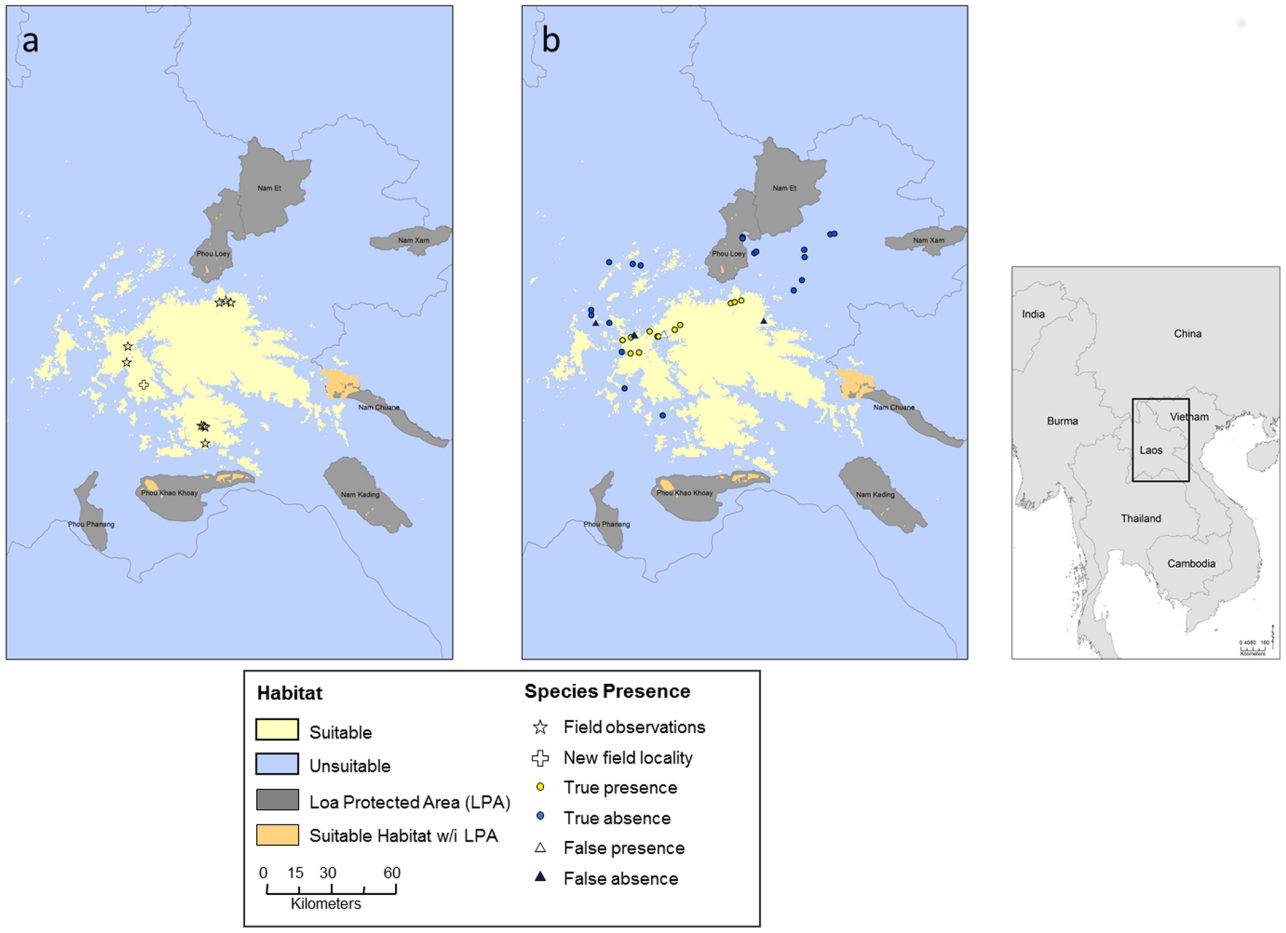
Binomial local Maxent model results. These results show habitat suitability for *L. laoensis* under a binomial classification of habitat as either suitable (good) or unsuitable (poor). The map was based on the results of the Full Model (see Figure 2a) with a threshold of 0.35. Lao protected areas are also shown in gray, and areas of suitable habitat that fall within the borders of an LPA are in orange. Figure (a) shows localities where *L. laoensis* was found during field surveys while (b) shows locations where interview data was used to identify the presence or absence of *L. laoensis.*

To further validate the model results, we first converted the continuous logistic output to a binomial map of good versus poor habitat. We specifically chose the Full Model at the local scale for validation because the local scale covered the entire extent of the species’ likely range and the Full Model used all available environmental data. Furthermore, as all models at the local scale were nearly identical in AUC (ranging from 0.969–0.970, Table 5), selecting the Full Model provides the greatest amount of environmental information without sacrificing model performance. The average lowest presence threshold for the Full Model was 0.35, so we used this value as the threshold for dividing suitable and unsuitable habitat (Figure 3).

Using this binomial map, we plotted the locations of both the presence data that went into the Maxent model (n = 9), and the additional sites for which we had interview data (n = 36). Given that the field survey locations were used to set the threshold of suitable versus unsuitable habitat, all presence points fell within suitable habitat (Figure 3a). For interview data, points that fell within the suitable habitat (i.e. a logistic value of 0.35 or greater) were considered predicted presence, while those that occurred in unsuitable habitat were considered predicted absence (Figure 3b). When comparing the numbers of points that fell within each category, we found very strong support for the predictive ability of the Maxent Model (Table 6). The model correctly predicted presence (n = 12) and absence (n = 19) at 31 of the 36 sites. The remaining five sites were divided between those where Maxent predicted presence but interviewees were unfamiliar with the species (false presence, n = 4) and those where Maxent predicted absence where interviewees were familiar with the species (n = 1). The Maxent predictions are significantly better than random at predicting presence or absence (χ^2^_(1, 36) = _15.97, p<0.0001).

**Table 6:** Contingency table of the 36 locations where *L. laoensis* presence or absence was determined by interview data.

		Species Known to Interviewees		
		Yes	No	
Species predicted present by Maxent model	Yes	12		4
	No	1		19

Each site fell in an area where Maxent predicted either presence or absence.

When we compared the results of the full model at the local scale using either just interview presence data or field locality data, we found high similarity (r = 0.854). When comparing the newly obtained field locality of *L. laoensis* to the predictions of the local scale model using field locality data, we found that this site occurred in an area of high suitability, with a logistic value of 0.63– well above our threshold of 0.35 (Figure 3a). We also re-ran the Maxent models including this new point, but found that the resultant maps and the percent contribution of the variables were nearly identical to the models that included the original nine localities (results not shown because the new models were indistinguishable from those shown in Figure 2, r = 0.998).

### Description of Suitable Environment

When evaluating the percent contribution of each environmental variable to each model, altitude, isothermality, and temperature seasonality were uniformly important in all the local scale models in which they were included (Table 7). In addition to these three variables, both temperature and precipitation contributed at least 5% to one additional local scale model (Table 7). Thus, we used these five variables to characterize suitable habitat using the variable response curves.

**Table 7:** The percent contribution of each environmental variable to each of the 5 local Maxent models run.

	% Contribution to Model				
Environmental Variable	Full Model	Reduced Model	Moderate Correlation (r<0.75)	Low Correlation (r<0.5)	Expert Opinion
Annual Mean Temperature	0.2	N/A	N/A	N/A	0
Mean Diurnal Range in Temperature	0	N/A	0	N/A	N/A
Isothermality	**23.4**	**23.5**	**22.8**	**43.1**	N/A
Temperature Seasonality	**30.6**	**32.1**	**31.2**	N/A	N/A
Maximum Temperature of Warmest Month	1.3	N/A	N/A	N/A	0.4
Minimum Temperature of Coldest Month	0	N/A	N/A	N/A	0
Temperature Annual Range	2.8	N/A	N/A	N/A	N/A
Mean Temperature of Wettest Quarter	0.3	N/A	N/A	N/A	N/A
Mean Temperature of Driest Quarter	0	N/A	N/A	N/A	N/A
Mean Temperature of Warmest Quarter	1.9	N/A	N/A	N/A	0.1
Mean Temperature of Coldest Quarter	0	N/A	N/A	N/A	**16.8**
Annual Precipitation	0	N/A	N/A	N/A	1
Precipitation of Wettest Month	0	N/A	N/A	N/A	N/A
Precipitation of Driest Month	0	N/A	N/A	N/A	N/A
Precipitation Seasonality	0.4	N/A	1.2	0.2	N/A
Precipitation of Wettest Quarter	0.6	N/A	1	N/A	N/A
Precipitation of Driest Quarter	0.1	N/A	N/A	N/A	N/A
Precipitation of Warmest Quarter	0.1	N/A	0.1	0	N/A
Precipitation of Coldest Quarter	0	N/A	0.5	**10.3**	N/A
Altitude	**38.3**	**44.3**	**43.2**	**46.4**	**81.7**

Values of N/A indicate variable that were not included in a particular model. Values over 5% have been bolded.

**Figure 4:**
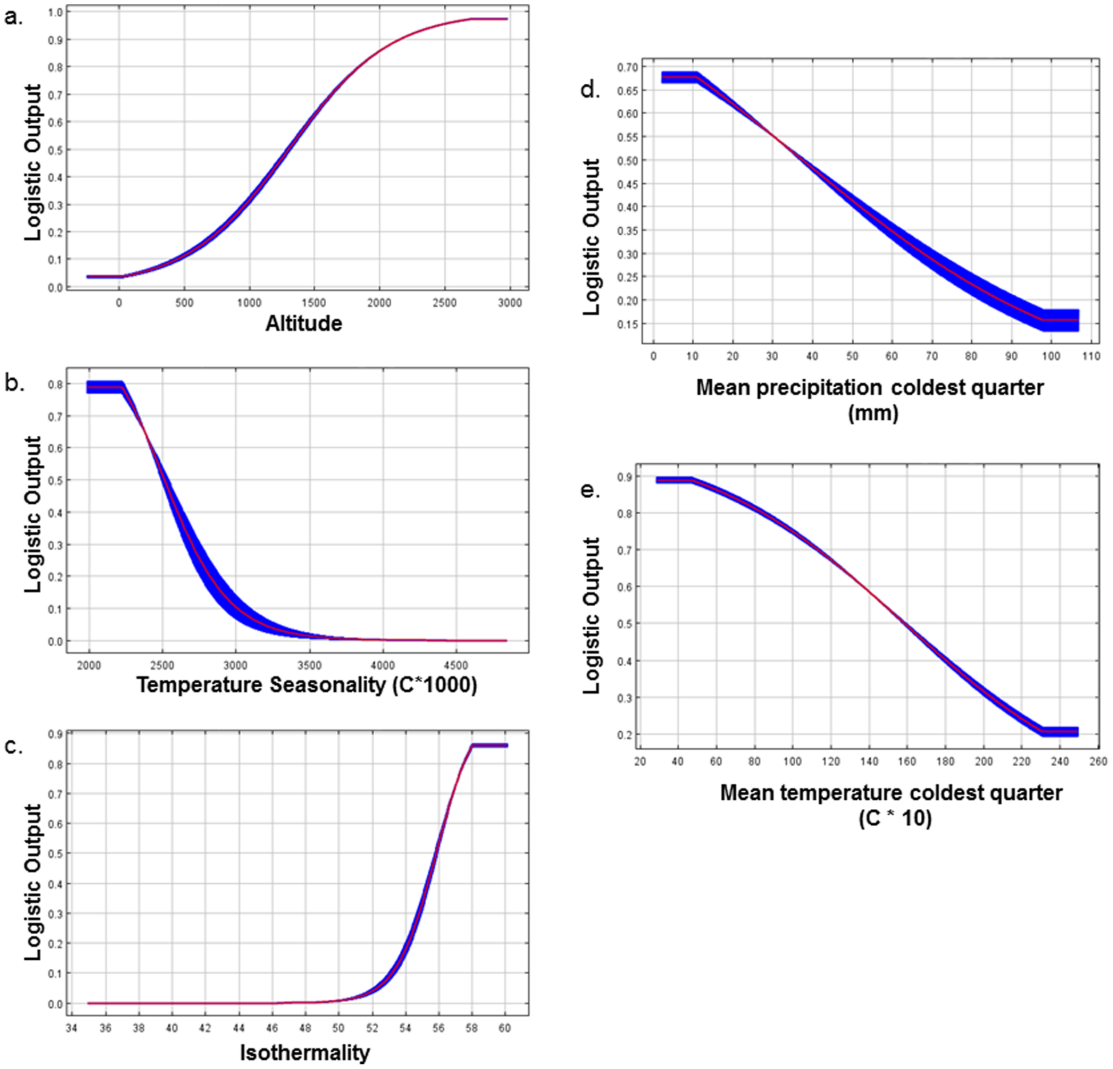
The variable response curves for the Full Maxent model at the local scale.

Altitude always had the highest percent contribution in each of the local scale models (Table 7). Although altitude is often strongly correlated with other environmental variables, it is an easy metric for characterizing habitat while in the field and thus it was useful to include here. The response curve for altitude showed a roughly linear increase in logistic output (and thus habitat suitability) with increasing altitude (Figure 4a). Suitable habitat for *L. laoensis* is therefore almost certainly restricted to high altitude sites over 1,000 m elevation, as concluded from field surveys [7].

The next two most important environmental variables in terms of percent contribution to the models were temperature seasonality and isothermality (Table 7). Both temperature seasonality and isothermality are a measure of the variability in temperature over the course of the year. Logistic output decreases with temperature seasonality (Figure 4b) and increases with isothermality (Figure 4c). Finally, mean precipitation of the coldest quarter and mean temperature of the coldest quarter each had a relatively high percent contribution to one of the local models (the Low Correlation model and the Expert Opinion model respectively, Table 7). The response curves show decreasing logistic output with increasing precipitation (Figure 4d) and temperature (Figure 4e).

**Figure 5:**
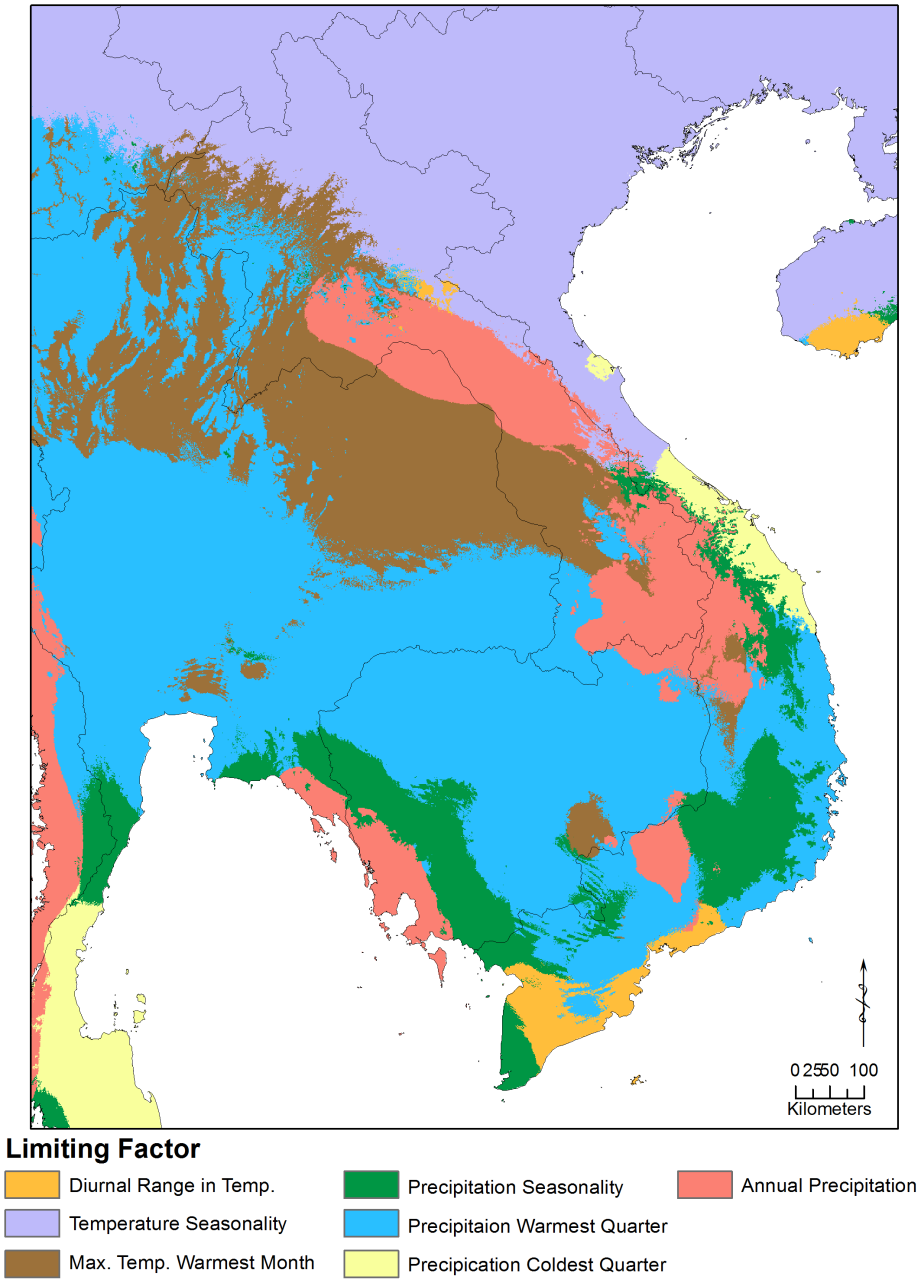
The limiting environmental factors identified at the regional scale.

**Figure 6:**
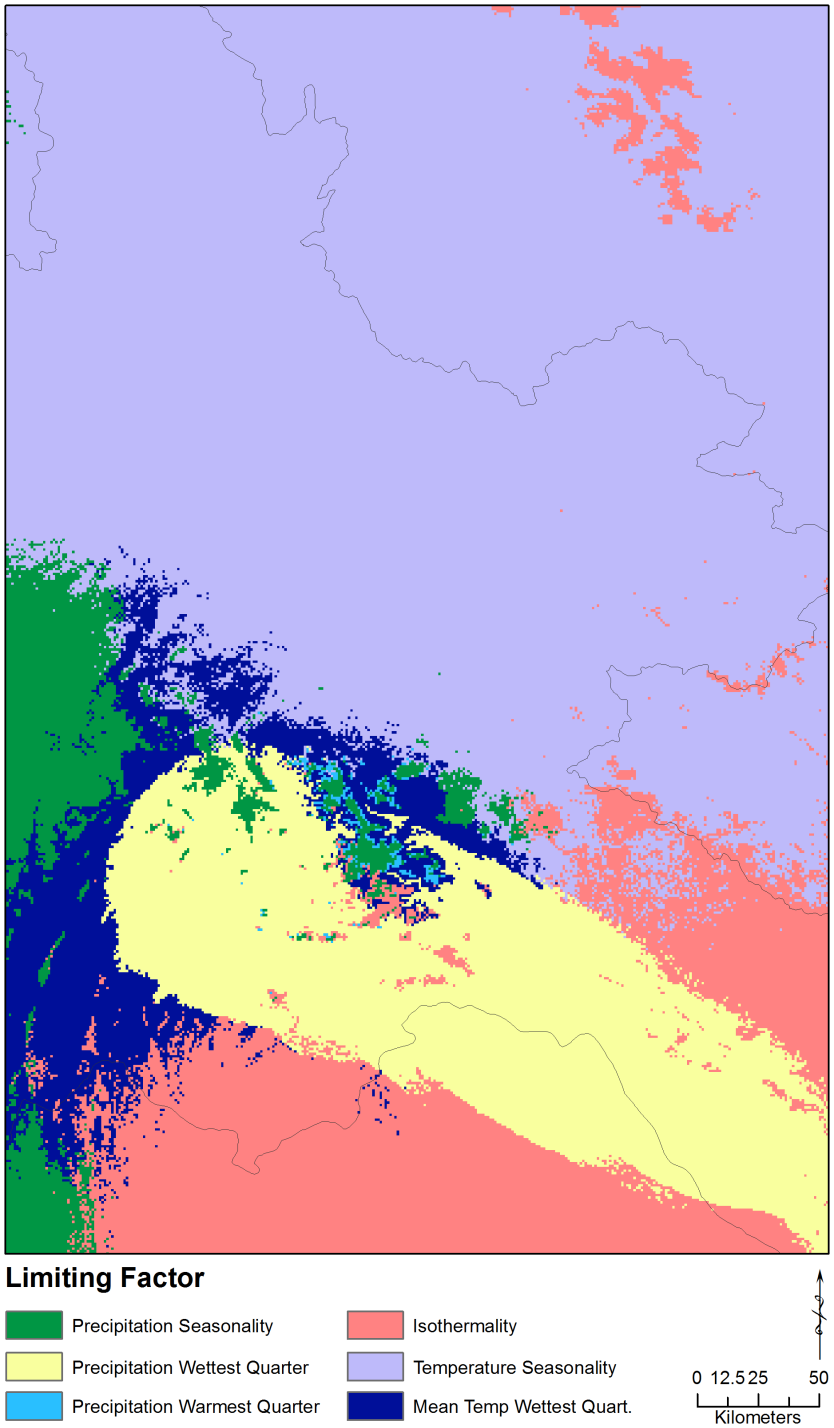
The limiting environmental factors identified at the local scale.

The limiting factors analysis shows that, at the regional scale, temperature seasonality, annual precipitation, maximum temperature of the warmest month, and precipitation of the warmest quarter are limiting in northern Laos (Figure 5). At the local scale, temperature seasonality and precipitation of the warmest quarter are still identified as limiting, along with precipitation seasonality, precipitation of the wettest quarter, temperature of the wettest quarter, and isothermality (Figure 6).

When we compared the distribution of suitable habitat for *L. laoensis* with the national protected area system of Laos, we found some suitable habitat patches in Phou Khao Khoay National Protected Area and Nam Chuane Proposed National Protected Areas (Figure 3).

When interpreting these data, it is important to consider that correlation between environmental variables is unavoidable in any study of this sort. Although we have minimized the issue of correlation between variables included in this model by selectively including variables that were only weakly correlated in the Moderate and Low Correlation models, it is possible that the variables reported as having a highly percent contribution to the model are not actually the drivers of the distribution of *L. laoensis*, but are instead important in the model only because those variables are correlated with environmental variables that were not included in the model. Regardless of whether these parameters directly shape the distribution of *L. laoenis* or are only correlated with the true drivers of the distribution, these results can still be used to identify likely areas for identifying other regions where *L. laoensis* may be found and provide a starting point for experimental work to elucidate the environmental factors most important in driving the realized niche.

## Discussion

Maxent is increasingly being used to identify important areas of conservation for amphibian species [14], [27]. Here, we used Maxent to predict the distribution of a poorly known, threatened species. This work highlighted the use of Maxent as a relatively inexpensive tool to study such species to establish priority sites for field sampling and implementing conservation measures. In particular, incorporating knowledge from local residents provided strong validation for our model results. While Maxent has been increasingly used in conservation research, model results are often difficult to validate with field work because of the multiple constraints inherent in conservation. Using local knowledge could be one method for validating results when field surveys are difficult to undertake.

We had several goals in modeling the distribution of *L. laoensis.* First, we established that this species is likely endemic to northern Laos, although a small region of predicted habitat was identified in Vietnam near the border with Laos. Although it is possible that the species could have a larger range than our models suggest, the fact that all models show a restricted range (regardless of area chosen and scale used), the models were validated by interview data, field work has only identified this species in the regions highlighted by the model, and the uniqueness of the species all suggest that this is a highly geographically restricted species. This finding has important implications for establishing conservation policies to manage this potentially endangered species. Preventing the extinction of this species in the wild will depend entirely on conservation efforts within Laos (if it does occur in Vietnam, it likely does so only marginally). Also, the endemic status of the species can be emphasized in education and outreach efforts to promote awareness and national pride in this unique species.

Second, we established that only limited areas of suitable habitat are currently protected. Only two national protected areas, Phou Khao Khoay in Laos and Nam Chuane in Vietnam, had moderate sized patches of suitable land. In both cases, these habitat patches are entirely isolated by a swath of unsuitable habitat from the regions where *L. laoensis* has actually been observed. It is therefore possible that the range of *L. laoensis* may fall entirely outside of established national protected areas. Field surveys of Phou Khao Khoay and Nam Chuane are urgently needed to determine if the species is found within these protected areas (although note that Nam Chuane remains only a proposed protected area and is not yet officially designated as such). If either protected area does support *L. laoensis*, an important next step will be to determine the rate of gene flow between protected and unprotected populations. Given that only a small fraction of the potential range is protected, and potential habitat in these protected areas occurs as isolated patches at the periphery of the range, establishing a new protected area within the core of the range should be considered an essential part of mitigating extinction of this species from overexploitation, habitat degradation, and climate change. Isolated habitat patches were also identified in Nam Kading and Phou Loey National Protected Areas. However, because of the small size and relative isolation of these patches, it is less likely that *L. laoensis* occurs within these protected areas.

Third, in characterizing the habitat, we confirmed that this species is restricted to high elevations (above 1,000 meters) and that altitude, seasonality, and precipitation and temperature of the coldest quarter of the year are particularly important variables in predicting the distribution of this species. Although these models identified correlation rather than causation, it is interesting to note that the coldest quarter of the year is also the *L. laoensis* breeding season ([7]). Laboratory experiments to establish the effect of temperature on survival and development at early life stages might provide important insights into the factors that limit the distribution of this species. The restriction to high altitudes also suggests that this species will be vulnerable to increasing temperatures in SE Asia, as the potential for the species to move to higher elevations is limited.

Finally, we aimed to identify areas for targeting future field surveys for this rare species. All of the Maxent models highlighted an area in northern Laos to the east of currently known populations as suitable for this species. Interview data strongly supported these model results. Only one site was found where Maxent predicted absence but interview data revealed that the species was actually present. This point is very close to predicted suitable habitat (Figure 3b). Four sites were predicted present by Maxent where this species was not recognized as present by interviewees. All four sites were found at the edge between suitable and unsuitable habitat suggesting that this might be marginal habitat for *L. laoensis*. Alternatively, a dispersal barrier would also explain the discrepancy between Maxent predictions and the actual distribution. Either way, the high predictive power of this model suggests that these results will be highly useful for guiding future field surveys. Furthermore, our use of interview data to validate model results provided a relatively inexpensive way to collect enough data to validate our model in a short amount of time while simultaneously engaging with the local community. We suggest that other studies could take advantage of this methodology to quickly provide data that can be used to construct and validate species distribution models, particularly for species that are reliably recognized by local residents in interviews and that need immediate conservation action.

*Laotriton laoensis* is currently at risk of extinction from several different causes. Previous work has demonstrated that this species is occasionally consumed for food and used for traditional medicine, and is often sold to commercial collectors for the international pet trade [6], [7]. In addition, our work here reveals that *L. laoensis* faces additional threats of a limited geographic range and a high altitude distribution rendering it vulnerable to climate change. It has previously been suggested that this species should be listed as Endangered by the IUCN and considered for listing by CITES [7]. This work reiterates the need for listing, as our results indicate that it is highly unlikely that this species will be found much beyond its currently known range. In addition, because suitable habitat is currently almost entirely unprotected, a new reserve should be established in core habitat to provide needed protection from harvesting and habitat degradation. Finally, our work serves as a case study of how modelers can work more closely with local people to create models that help to efficiently focus resources toward on the ground conservation.
